# *Shigella* Draft Genome Sequences: Resources for Food Safety and Public Health

**DOI:** 10.1128/genomeA.00176-17

**Published:** 2017-04-20

**Authors:** Allison M. Weis, Brent Gilpin, Bihua C. Huang, Nguyet Kong, Poyin Chen, Bart C. Weimer

**Affiliations:** a School of Veterinary Medicine, 100K Pathogen Genome Project, UC Davis, Davis, California, USA; b Institute of Environmental Science & Research Ltd., Christchurch, New Zealand

## Abstract

*Shigella* is a major foodborne pathogen that infects humans and nonhuman primates and is the major cause of dysentery and reactive arthritis worldwide. This is the initial public release of 16 *Shigella* genome sequences from four species sequenced as part of the 100K Pathogen Genome Project.

## GENOME ANNOUNCEMENT

*Shigella* spp. are Gram-negative enteric pathogens that infect humans and nonhuman primates. They are an important cause of dysentery, affecting more than 80 million people and causing more than 700,000 deaths each year worldwide (1, 2). The burden of disease is carried by children, where 99% of infections occur in children in developing nations, and most cases (70%) and deaths (60%) occur in children age 5 and under (1, 2). Rare cases of shigellosis can lead to reactive arthritis (3). *Shigella* is spread by direct contact with an infected person or by ingesting contaminated food or water (1, 4). The infective dose can be as few as 10 organisms, making *Shigella* a foodborne pathogen of global importance based on wide distribution, water quality concerns, and an important risk for public health (4).

The genus *Shigella* is composed of four species: *S. dysenteriae*, *S. flexneri*, *S. boydii*, and *S. sonnei*, all of which cause acute bloody diarrhea (2, 5). *Shigella* genomics has emerged as an important tool in basic and clinical applications for diagnosis and classification, and will inform treatment plans (5, 6), but the ability to conduct source tracking using whole-genome sequencing remains challenging due to the relatively few publically available genomes. In this release, the 100K Pathogen Genome Project sequenced and assembled the genomes of 16 novel *Shigella* isolates of the four species: two* S. boydii*, three *S. dysenteriae*, nine* S. flexneri*, and two* S. sonnei* isolates (Table 1).

**TABLE 1:** *Shigella* species draft genome sequence information

GenBank accession no.	Strain ID	Species	Depth (×)	No. of contigs	No. of bases
MSJS00000000	BCW_4868	*S. boydii*	115	243	4,863,576
MSJT00000000	BCW_4869	*S. boydii*	108	297	4,246,029
MSJU00000000	BCW_4870	*S. dysenteriae*	114	285	4,018,103
MSJV00000000	BCW_4871	*S. dysenteriae*	117	299	4,078,019
MSJW00000000	BCW_4872	*S. dysenteriae*	72	292	4,490,659
MSJX00000000	BCW_4874	*S. flexneri*	109	269	4,252,909
MSJY00000000	BCW_4875	*S. flexneri*	90	249	4,196,256
MSJZ00000000	BCW_4876	*S. flexneri*	101	293	4,396,898
MSKA00000000	BCW_4877	*S. flexneri*	100	296	4,330,224
MSKC00000000	BCW_4879	*S. flexneri*	124	287	4,167,963
MSKB00000000	BCW_4880	*S. flexneri*	106	267	4,224,783
MSKD00000000	BCW_4881	*S. flexneri*	170	253	4,334,622
MSKG00000000	BCW_4882	*S. flexneri*	96	297	4,099,589
MSKF00000000	BCW_4883	*S. flexneri*	118	289	4,305,926
MSKE00000000	BCW_4885	*S. sonnei*	101	299	4,392,417
MSKH00000000	BCW_4886	*S. sonnei*	100	286	4,530,575

The 100K Pathogen Genome Project (http://www.100kgenomes.org) is a large-scale sequencing effort to inform food safety and public health in genome-based identification and source tracking (7, 8). All *Shigella* isolates were shipped to Bart Weimer’s laboratory (UC Davis, Davis, CA). DNA isolation, sequencing, and assembly were done as previously described (7–9). Briefly, isolates were checked for purity (10) prior to extracting genomic DNA (gDNA) from cultures grown on brain heart infusion agar (catalog no. 241830; BD Difco, Franklin Lakes, NJ) for 1 to 2 days at 37°C. Cells were lysed (11), gDNA was purified using the Qiagen QIAamp DNA minikit (catalog no. 51306), and quality was measured using the Agilent 2200 TapeStation system with the Genomic DNA ScreenTape (12). After isolation, gDNA was fragmented using Covaris E220 (13), end-repaired (5′), adenylated (3′), and ligated with double-stranded DNA (dsDNA) adapters NEXTflex-96 DNA barcode (Bioo Scientific, Austin, TX), and gDNA (1 μg) was used for library construction with the Kapa high-throughput (HTP) library preparation kit (catalog no. KK8234; Kapa Biosystems, Boston, MA), using the Agilent Bravo automated liquid handling platform workstation option B (Santa Clara, CA). The libraries were size selected using dual SPRI selection (0.2× to 0.6×) to produce libraries with fragments between 300 and 450 bp. Final library amplification was done with eight cycles using the Kapa HiFi HotStart ReadyMix, followed by a 1× SPRI bead cleanup. Prior to sequencing, the library size was confirmed using the Agilent 2100 Bioanalyzer system with high-sensitivity DNA kit (14, 15), quantified with a quantitative PCR (qPCR)-based Kapa library quantification kit (catalog no. KK4824), pooled with multiplexing up to 96 isolates, and sequenced on the Illumina HiSeq 2000 with PE100 plus index read at BGI@UC Davis (Sacramento, CA). The paired-end reads were assembled using CLC Genomics Workbench version 6.5.1 (Qiagen).

### Accession number(s).

Sequences can be found in the NCBI SRA 100K Project BioProject PRJNA186441 and in GenBank (Table 1).
